# Courage, Fortitude, and Effective Leadership of Surgical Teams During COVID-19

**DOI:** 10.1177/2150135120938330

**Published:** 2020-07-10

**Authors:** Elizabeth H. Stephens, Joseph A. Dearani, Kristine J. Guleserian

**Affiliations:** 1 Department of Cardiovascular Surgery, 4352 Mayo Clinic, Rochester, MN, USA; 2 Department of Congenital Heart Surgery, Medical City Children’s Hospital, Dallas, TX, USA

**Keywords:** COVID-19, wellness, mental health, cardiothoracic surgery

## Abstract

The world as we once knew it has been drastically altered secondary to coronavirus disease 2019 (COVID-19). The impact of these changes, particularly for those practicing in the medical profession, extends beyond the physical to the psychological, emotional, and spiritual. We discuss the factors that contribute to these stresses, way to manage them, and how we as leaders of our teams can inspire resilience and help our colleagues endure these most difficult times.

## Introduction

One of the dangers of traumatic and world-changing events such as 9/11—*and now the COVID-19 pandemic*—is that they can trigger sudden, intense feelings of helplessness and hopelessness. Physician and health care workers’ physical safety has been the primary focus early on in this crisis and is essential to managing the ongoing pandemic. However, our psychological, emotional, and spiritual well-being are also key to performing under stressful conditions unlike any we have encountered before. Even prior to the coronavirus disease 2019 (COVID-19) pandemic, many clinicians and health care workers were experiencing burnout, as well as stress, anxiety, fatigue, and depression. Some experience suicidal thoughts, or turn to substance abuse. The population of health care professionals prone to such experiences extends to those without any underlying or preexisting mental health conditions. Today there are not only workplace hardships imposed by the pandemic, but also unprecedented ethical and moral dilemmas that are likely to create or exacerbate existing levels of burnout and mental health-related problems. In contrast to other individually experienced traumatic events when those around us have the emotional reserve to support us, during COVID-19 all members of society are under immense strain, albeit in different manners. Based on experience in other outbreaks, the psychological burden may last long after this pandemic is over.^1,2^ In this commentary, we discuss the factors that test our emotional resilience during this time and how we, as leaders of our surgical teams, can foster fortitude in ourselves and those around us, to ultimately provide the best care possible for our patients.

Although the terms “wellness” and “mental health” are commonly used in describing these issues, we prefer the term “fortitude.” Fortitude is defined as mental and emotional strength in the face of difficulty or adversity.^3^ Although we all strive for peak performance, various nonphysical factors—*a conglomeration of mental, emotional, and psychological*—can prevent us from doing exactly that. Recognizing, grappling with, and harnessing these factors to our advantage is critical during these unprecedented times.

## Baseline Challenges to Fortitude in Cardiothoracic Surgery

Cardiothoracic surgery is a high-risk high-reward specialty with one of the lengthiest postgraduate training paradigms followed by many years at the faculty level to attain a high level of technical expertise and judgment. Any lack of technical precision or lapse in attention to detail during an operation may have drastic implications for our patients. Thus, our specialty puts us at risk for emotional distress and anxiety on a daily basis. Many factors contribute to this: time demands, the life and death nature of the operations for every patient with little margin for error, expectations to always appear and perform at “the top of our game,” and the strain of managing our personal lives. With the addition of external events such as COVID, or professional and personal issues, we are a group of professionals whose fortitude is at risk. Beyond these factors, we have been conditioned through our training to constantly perform self-assessment, be receptive to critique, and aim to continuously improve. As popularized by Gladwell and Ericcson,^4,5^ those who excel at highly technical tasks largely do not attain such heights by ability alone, but via a long-standing, continual process of practice, critique, and improvement. Although this produces superior performance, it also may ingrain in us elements of perfectionism that can be harmful. Similarly, growing up in the culture of cardiothoracic surgery fosters an image of indefatigability that becomes a badge of honor.^6^

It has become increasingly apparent that cardiothoracic surgeons experience anxiety, depression, and burnout that may prevent us from achieving peak performance.^7^ Results from the recent Society of Thoracic Surgeons (STS) practice survey demonstrated that nearly 60% of respondents had feelings of burnout at least a few times a month in the last year^8^…the stress is real.

## Additional Stressors Related to COVID-19

The COVID pandemic adds a whole new set of stressors to our profession that is already vulnerable. During the peak of the COVID-19 pandemic, surgeons were operating much less, taking us from our daily routine of surgery. Our routines confer a sense of purpose, value, predictability, and for many, a sense of control and comfort—after all, we pursued this career because we love to operate and thrive on challenges. The cancellation and rescheduling of cases presented us with exceedingly difficult ethical dilemmas in terms of prioritizing those that can and should be done during justified proscription of “elective” surgeries. Who should be offered surgery and when should surgery be done replaced our routine surgical scheduling mindset that was nearly automatic and taken for granted. Additional work-related challenges included physical distancing and isolation in some circumstances. And in the setting of reactivation, there is a perceived demand at many institutions to substantially increase volume over original baseline to “catch up” and operate on patients who have been postponed and all of those currently in need. Striking the right balance between safe and complete recovery of services versus the potential increase in viral spread if screening, personal protective equipment (PPE) and physical distancing protocols are not adhered to, contributes substantially to added tension. Finally, while high-quality patient care and safety remains the highest priority, the favorable financial portfolio of the cardiovascular service line to a hospital adds additional pressure to the surgical teams to perform cases.

An important undercurrent of this pandemic and source of stress for the cardiothoracic surgeon is the *uncertainty of the present and future*. As surgeons who are most comfortable being in control, our personal and professional lives can feel out of control when uncertainty is present—uncertainty regarding the timeline of reactivation, duration and safety of delay for patients, the constantly changing screening protocols, what protective equipment is most appropriate and what is available, the timing and magnitude of resurgence(s), and the future health of ourselves, our team members, and our families. We constantly strive to do what is best for our patients who need time-sensitive surgery, weighing the risks and benefits of proceeding versus delaying. But what the risks and benefits are during these times, and what is actually best, remains abundantly *unclear*—a muddy collage of various shades of gray, with no guidelines in textbooks or papers to turn to, and no “levels of evidence” at this time.

One recent study based on the COVID experience in China identified that the most impactful factors associated with stress among health care workers were personal safety, concern for their families, and patient mortality.^9^ In another study from China, the biggest factors were loss of control, vulnerability to infection, fear for personal health, and spread of the virus.^10^ Studies have shown that the mental health burden during COVID is considerable. In a study by Lai et al, 50% demonstrated depression, 45% anxiety, and 34% insomnia, with frontline workers at highest risk.^11^ So, the stress is real.

## Personal Stressors Related to COVID-19

COVID-19 not only adds a considerable burden of strain related to work, but also impacts our personal lives. First, how to minimize the risk and impact of exposure experienced by our families as a result of our work at the hospital—whether that be in the form of a protocol for “decontamination” before interacting with family when returning from work, or sheltering alone. For some, children are now home and having to be homeschooled. With another working parent who is trying to juggle a career from home, this is exceedingly difficult. For others with young children, the previously utilized childcare is no longer available. Some have been faced with trying to assist and care for elderly parents, or other family members with disabilities, often remotely. A spouse or partner may be out of work, adding additional financial and emotional tension. And still others are faced with social distancing while living alone, which is an added strain. Many conveniences that we relied on are not available, whether that be care for our home, our pets, or obtaining basic necessities. The simple act of going to the grocery store with the potential for infection transmission from either people density or significant hand contact with objects (ie, groceries, carts, etc), or both, accounts for a whole new level of incalculable risk and essentially unavoidable stress. The expectation of wearing masks in public places, handwashing, and physical distancing seems to have no end in sight, adding enormous strain. For all of us, we are worrying and trying to care for our families and loved ones in the midst of a pandemic that is particularly dangerous for certain “at risk” populations…and nearly every person has one or multiple family members who fall into this category…the stress is real.

## Consideration of the Care Team and Patients

The stressors of COVID not only involve our work lives and personal lives but extends throughout our entire hospital care team and our patients, thereby further impacting us and our ability to provide optimal patient care. Everyone in the workplace has been and continues to be affected, all members of the surgical team in the operating room, intensive care unit, wards—from surgeons to secretaries. Team management for some has changed to shift work of alternating teams, with some staying at home and others working remotely. Many of our nonclinical team members are working remotely at home, which adds a myriad of additional strains—less efficiency for those at work and more distractions for those at home. There is lack of work–home structure and boundaries, lack of the work infrastructure, and professional and personal support systems. Added into this mix are elements such as homeschooling for children or needing to provide childcare while also working, or remotely caring for parents/grandparents who are at increased risk. Many are facing substantial financial strain, which can range from pay cuts among other family members no longer generating income. For families with lower socioeconomic status, some children relied on school lunch meals that ceased to be available, adding more personal stress. Furlough is particularly difficult for members of our teams as it cuts off their professional role and value, disrupts the camaraderie and support system of work, and eliminates needed income. The stress felt by our patients and their families is another important component, particularly in cardiothoracic surgery where some risk of mortality, albeit small in most cases, is always present. Whether it be the restrictions imposed on families limiting their presence with patients during a hospital stay, to difficulty communicating with and supporting their loved ones, to worrying about contracting COVID, to their own financial stress, the additional burden on our patients and their families also impacts us, making the stress and anxiety tangible.

## Effective Team Leaders

As cardiothoracic surgeons, we are in many respects the natural leaders of our respective teams and the ones to set the tone and example for others. Communication and fostering connectivity during this time of crisis is crucial. Weekly leadership discussions and town halls, conference calls, large interactive webinars, and/or smaller scale virtual meetings using WebEx/Zoom-type platforms are facilitating our teams’ ability to stay informed. They also enable members to relay their evolving needs and concerns and us to relay ours. Listening to our team members and specifically asking them about their concerns and needs is vital to such discussions. Acknowledging their stresses and recognizing that previously simple tasks are now more complex can be particularly reassuring and meaningful. Although we are all physically distant, remaining connected intellectually and emotionally is critically important. Sincere gratitude from leaders and between coworkers can be a powerful source of support. In this time of change, our leadership styles and strategies must evolve in response to the changing needs of our team, but in all circumstances, we should lead by example.

## Action Items—Fostering Fortitude

The first step in fostering fortitude in ourselves and our teams is recognition of the increased stress and the risk of declining wellness during this time. The second step is learning to recognize and acknowledge symptoms of diminishing fortitude, such as fatigue, fear, anxiety, depersonalization/cynicism, emotional exhaustion, low sense of accomplishment related to work, withdrawal, and guilt.^7,12^ And the third step is developing habits to improve fortitude. Maddaus has identified key habits that improve a surgeon’s resilience: sleep, exercise, mindfulness and meditation, gratitude, self-compassion, and connection to others.^13^ In the review by Fann et al, factors promoting resilience encompass individual, family, organizational, and community components and an adapted list is provided Table 1.^7^ Other studies have similarly identified exercise, focusing on what is important in life, vacations, and nurturing one’s religious/spiritual life as protective against burnout.^14^ Certain personality traits, such as being grounded in reality, having a robust value system, and the ability and willingness to improvise when faced with challenges are also correlated with resilience.^15^

**Table 1.** Factors Promoting Resilience.^a^

Individual	Family	Organizational	Community
Flexibility, ability to innovate	Adaptability	Positive command climate	Belongingness
Positive affect and thinking, gratitude	Communication	Teamwork	Cohesion
Remaining grounded in reality	Providing and seeking support	Cohesion	Connectedness
Focusing on what is important	Closeness	Expressing appreciation to team members	Collective efficacy
Concentrating on what can control		Acknowledging stress in others	Altruism
Mindfulness and meditation		Listening, providing opportunities for feedback	
Nurturing religious/spiritual beliefs		Active, bidirectional communication	
Altruism			
Sleep			
Physical fitness			

^a^ Adapted from the study by Squiers et al^7^ and Meredith et al.^16^

Specifically within pandemics including COVID, studies in Chinese health care workers have identified optimism and altruism as elements that may decrease psychological strain.^17,18^ In the setting of COVID, practical measures such as providing PPE and clear practice guidelines were shown to decrease stress, as was appreciation of their work.^2,19^ Another recent survey of the experience in China identified correct guidance, safeguards against transmission, positive attitude of staff, and safety of family members as having the biggest impact on stress of staff members.^9^ Positive coping mechanisms included strict protective measures, knowledge of viral spread, and a positive self-attitude, with seeking help from family and friends also cited as important.^9^ As leaders, we can aid in this by providing clear communication and guidelines, implementing safeguards against transmission, maintaining a positive attitude, and managing our stress adequately, while expressing appreciation for our team’s work and dedication.

However, as Stanford Medicine’s chief wellness officer, Tait Shanafelt, MD, stated “We should not be recycling the wellness offerings of the past, as if retooled versions of those approaches are the current needs…we need to approach this situation with fresh eyes, ask our people what they need, develop our response based on the needs they’ve expressed, and effectively and compassionately communicate with them.”^20^ Part of approaching this topic differently during COVID is the platform—gyms and yoga studios are closed, social distancing does not allow for coffee with a friend, those in densely populated areas cannot enjoy parks, and other support systems that depend on gatherings, such as religious services, are on hold. During this time, we rely on technology to keep us connected and have to be creative in redefining stress-relieving activities we once enjoyed or create new ones. Of course, ensuring and encouraging adequate and appropriate mental health care when needed may help physicians and health care workers develop improved emotional and cognitive resilience to withstand the impact of such traumatic events.

## Conclusion

Times such as this pandemic test our fortitude. Cardiothoracic surgery is the prototype specialty that has a complex interface between patient and technology, and seamless, effective teamwork is necessary for good outcomes. As cardiothoracic surgeons, we are innate leaders. The cardiothoracic surgeon has courage by nature and a “never give up” attitude. In a specialty where every day is “game seven” and the expectation is perfect performance each and every time, the stress level is already high. Similar to the technical (physical) skills and judgment that we have developed over the years to become excellent surgeons, cultivation of skills related to emotional resilience are also critical to ensure that we maintain competence and compassion for our patients, our teams, and our families, while upholding our uncompromising demand for excellence.

As Abigail Adams wrote to her son John Quincy Adams during the American Revolution “It is not in the still calm of life, or the repose of a pacific station, that great characters are formed” rather that “the habits of a vigorous mind are formed in contending with difficulties. Great necessities call out great virtues.”^21^ The importance of our leadership in this most turbulent time, including the fostering of fortitude in us and others, should not be underestimated.
